# Editorial Note: The role of 9-*O*-acetylated glycan receptor moieties in the typhoid toxin binding and intoxication

**DOI:** 10.1371/journal.ppat.1014413

**Published:** 2026-07-13

**Authors:** The PLOS Pathogens Editors

The *PLOS Pathogens* Editors issue this Editorial Note to inform readers that, after this article [1] was published, concerns were raised that the SPR data presented in Figs 3B-F appear to be fitted, exported curves rather than raw SPR data.

The corresponding author stated that, as noted in the Materials and methods of [1], the SPR data presented in Fig 3B-F are fitted and exported curves from the Tracedrawer® software which is part of the OpenSPR® data viewer and processor. They provided the raw SPR data underlying Figs 3B-F in S1 File.

A member of the *PLOS Pathogens* Editorial Board stated that the fitted curves match the raw data well and that the interpretation of the data is adequate.

## Supporting information

S1 File The raw SPR data underlying Figs 3B-F, including: combined graphs of raw sensograms and fitted curves for all glycans tested; combined fitted curves for all glycans tested; extracted kinetic values from Tracedrawer® software; the Tracedrawer® software project file with all graphs, curve fittings, and kinetic value outputs; text files of raw data points and fitted curves for each glycan tested; Excel files of individual glycan data fitted into graphs; and exported continuous data point outputs from the OpenSPR instrument for each glycan group containing the entire response signal (experiment start – chip cleaning – protein loading – wash and glycans concentration injections– wash & clean – chip removal). (ZIP)
